# Applications and Prospects of Single-Cell RNA Sequencing and Spatial Transcriptomics in Cervical Cancer

**DOI:** 10.1155/bmri/1532745

**Published:** 2025-06-26

**Authors:** Yifu Wang, Li Yang, Yunzhi Liu, Huangrong Ma, Miaoying Cai, Chunyu Liang, Li Zhang, Zezhuo Su, Zhiyuan Xu

**Affiliations:** ^1^School of Medicine, Shenzhen University, Shenzhen, Guangdong, China; ^2^Clinical Oncology Centre, The University of Hong Kong-Shenzhen Hospital, Shenzhen, Guangdong, China; ^3^Department of Radiology, The University of Hong Kong-Shenzhen Hospital, Shenzhen, Guangdong, China; ^4^Obstetrics and Gynecology, The University of Hong Kong-Shenzhen Hospital, Shenzhen, Guangdong, China; ^5^Department of Orthopaedics and Traumatology, School of Clinical Medicine, Li Ka Shing Faculty of Medicine, The University of Hong Kong, Pokfulam, Hong Kong SAR, China

**Keywords:** cervical cancer, single-cell RNA sequencing, spatial transcriptomics, therapeutic targets, tumor microenvironment

## Abstract

Cervical cancer (CC) is the fourth commonest malignant tumor among women worldwide and is characterized by high heterogeneity and a complex ecosystem. A comprehensive understanding of the heterogeneity of tumors and the tumor microenvironment (TME) is crucial for effective CC management. Single-cell RNA sequencing (scRNA-seq) is a powerful tool that can be employed to unveil the heterogeneity of tumors and the TME, as well as to elucidate the evolutionary trajectories of tumors. Spatial transcriptomics (ST) technology, on the other hand, can address the complexity and diversity of the spatial microenvironment of tumors, thereby compensating for the limitations of scRNA-seq. As emerging technologies, both scRNA-seq and ST are increasingly being utilized in CC research. In this review, we summarized the latest advancements in scRNA-seq and ST for CC, with a focus on investigating tumor heterogeneity, the TME, tumor evolutionary trajectories, treatment resistance mechanisms, and potential therapeutic targets. These insights collectively contribute to the development of more effective treatment and prevention strategies for CC.

## 1. Introduction

Cervical cancer (CC) is one of the commonest gynecological malignant tumors in women and is also one of the four leading causes of cancer-related deaths in women [1]. Unlike most common cancers, the survival rate for women with malignant cervical tumors has not significantly improved over the past 40 years, largely reflecting the lack of significant therapeutic advancements [2]. The main treatment methods for CC include surgery, radiotherapy, chemotherapy, and immunotherapy [3]. Nearly half of newly diagnosed CC patients are identified as having locally advanced disease, for which the standard treatment involves concurrent chemotherapy and radiation therapy (CCRT). Owing to the development of treatment resistance, advanced CC poses significant challenges for effective management [3]. Exploring the molecular mechanisms of treatment resistance and discovering new therapeutic targets are highly important for CC treatment.

The tumor microenvironment (TME)—which consists of immune cells, fibroblasts, and extracellular components—plays a crucial role in the progression of CC and its response to therapy [4]. Tumor heterogeneity encompasses the wide-ranging molecular, genetic, and epigenetic variations within a tumor. These variations include differences in DNA mutations such as somatic mutations and copy number alterations, epigenetic modifications like DNA methylation and histone acetylation, distinct gene expression profiles, and diverse functional behaviors among various subpopulations of cancer cells. These variations fuel the initiation, progression, metastasis, and therapeutic resistance of tumors. This complexity is further exacerbated by the intricate and dynamic interactions with the TME, rendering heterogeneity a critical determinant of treatment outcomes [5]. Therefore, elucidating tumor heterogeneity and the TME—including their dynamics following treatment—provides vital insights for optimizing CC therapy. In recent years, the rapid development of single-cell RNA sequencing (scRNA-seq) technology and its widespread application in cancer research have significantly improved our understanding of the heterogeneity within the tumor ecosystem, thereby offering broad prospects for the development of novel therapies [6]. Meanwhile, the emerging technology of spatial transcriptomics (ST) can address the complexity and diversity of the spatial microenvironment of tumors, thereby compensating for the spatial resolution limitations of scRNA-seq [7, 8].

In this review, we summarize the latest advancements in the application of scRNA-seq and ST in CC researches, with a focus on investigating tumor heterogeneity, the TME, tumor evolutionary trajectories, mechanisms of treatment resistance, and potential therapeutic targets. The insights gained from these studies will enhance our understanding of CC, aid in the identification of potential therapeutic targets, offer new treatment strategies, and ultimately advance the treatment and prevention of CC.

## 2. Single-Cell Sequencing Technology

Single-cell sequencing technology encompasses genomic, transcriptomic, and proteomic sequencing. It identifies genetic variations and gene expression differences between cells at a high resolution. This enables researchers to gain a deeper understanding of the characteristics of individual cells, making it a highly influential technology [9, 10]. scRNA-seq is the most widely used single-cell sequencing technology. Its primary steps include single-cell isolation and extraction, cDNA synthesis, nucleic acid amplification, sequencing, and data analysis [11]. These main procedures have been extensively applied and demonstrated in other studies [12].

## 3. ST

In the workflow of single-cell sequencing, the preparation of single-cell suspensions leads to the loss of spatial information about the cells' original positions within tissues. Traditional techniques such as immunohistochemistry (IHC) and in situ hybridization provide high spatial resolution but are not well suited for high-throughput exploratory analysis [13, 14]. To address the spatial resolution limitations of scRNA-seq, ST was developed by Ståhl et al. in 2016 [7]. This technique provides both spatial resolution and transcriptomic expression information, making it ideal for analyzing the cellular composition and spatial distribution within tumors. It also enables the construction of spatial trajectories and interactions based on this information [7, 15]. By combining scRNA-seq with ST, it becomes possible to visualize and conduct quantitative analysis of entire tissues at a spatially resolved transcriptome level. This approach preserves both the cellular spatial distribution and single-cell resolution. In recent years, the integration of scRNA-seq and ST has emerged as a cornerstone in studying the immune microenvironment and cellular heterogeneity across various solid tumors [7, 15, 16]. The advent of ST has marked the beginning of the era of high-throughput spatial sequencing of large tissue areas. In the coming years, research into the cellular microenvironment and spatial heterogeneity is poised to advance rapidly.

## 4. Application of scRNA-seq in CC

As a rapidly evolving technology, scRNA-seq has been increasingly used in CC research. We have summarized the recent applications of scRNA-seq in CC in Table 1.

### 4.1. Decoding Tumor Heterogeneity in CC

CC exhibits extensive heterogeneity, which manifests as the presence of diverse cell types and biological properties within a tumor. These differences can be observed at both the genetic level and in cellular phenotypes. Li et al. used scRNA-seq to analyze the transcriptomes of 57,669 cells derived from three CC samples. Their analysis revealed extensive heterogeneity among malignant cells in human CC, with distinct genomic and transcriptomic features observed across different epithelial subgroups [31]. Here, we will delve into the heterogeneity that arises during the progression from normal cervical development to CC. Additionally, we will examine the two primary histological types of CC—squamous cell carcinoma (SCC) and adenocarcinoma (ADC)—as well as the unique characteristics of human papillomavirus (HPV)–associated CC.

#### 4.1.1. Molecular Divergence Between SCC and ADC

The main histological subtypes of CC are SCC and ADC. Despite their distinct histological features, detailed comparisons of tumor heterogeneity between SCC and ADC of cervix remain relatively underexplored in the literature [27]. Single-cell analyses have unveiled distinct molecular profiles between SCC and ADC of the cervix. For instance, Li et al. compared tumor cells from patients with SCC and ADC of the cervix. They revealed that SCC of the cervix is characterized by upregulation of immune-related pathways such as epithelial–mesenchymal transition (EMT), hypoxia, and inflammation, whereas ADC of the cervix shows enrichment in cell cycle pathways, including MYC targets and the G2M checkpoint [27]. Further studies by Qiu et al. confirmed these findings, demonstrating that SCC-specific markers (e.g., *S100A8* and *KRT14*) and ADC-specific markers (e.g., *LGALS4* and *CLDN3*) could effectively stratify subtypes and guide therapeutic targeting [22]. Additionally, distinct epithelial subclusters (Epi1–Epi6) were identified, each associated with unique pathway activation profiles: Epi1 (ADC-like) exhibited activation of hypoxia and oncogenic signaling pathways; Epi2 and Epi4 showed metabolic reprogramming; and Epi5 (SCC-like) was characterized by EMT pathways [30]. These findings underscore the subtype-specific vulnerabilities and identify potential biomarkers for precision therapy.

#### 4.1.2. HPV-Driven Tumor Cell Heterogeneity

To study the intratumor heterogeneity between HPV-associated and HPV-independent cancer, Li et al. used scRNA-seq to investigate the differentiation-related heterogeneity of viral genes and host cells in HPV-associated CC tissue. The clusters containing the highest proportion of HPV-associated cells in the epithelium exhibited deviations from normal epithelial markers, revealing functional heterogeneity and alterations in epithelial structure. This finding indicates substantial molecular heterogeneity within the cancer tissue, with these HPV-associated cells displaying different gene expression profiles compared to normal epithelial cells. Specifically, compared to HPV-independent cells, the HPV-associated cells exhibited unique gene expression patterns related to the extracellular matrix, cell adhesion, proliferation, and apoptosis [34].

### 4.2. Deconstructing the TME in CC

The TME plays a critical role in the occurrence, progression, invasion, metastasis, and treatment resistance of tumors [37]. Researchers believe that understanding the characteristics of the TME can aid in understanding the interactions between the TME and cancer cells, as well as in developing new strategies for cancer treatment [5, 38]. The features of the TME include immune cells, stromal cells, cancer cells, blood vessels, and the extracellular matrix. Among these features, immune cells are key factors in the TME and play a crucial role in the occurrence and treatment of tumors [39]. scRNA-seq technology can help researchers explore rarer and more heterogeneous cell populations within the TME [40].  Table 2 summarizes the cellular subsets in the currently published literature related to single-cell sequencing for CC.

**Figure 1:**
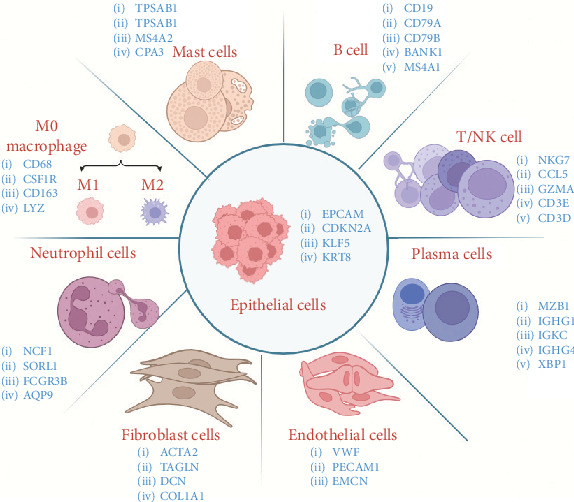
Cell types and markers in cervical cancer. In single-cell sequencing of cervical cancer, cell subgroups are typically clustered, with blue labels indicating the marker genes for each subgroup.

As shown in Table 2 and Figure 1, based on the published single-cell sequencing literature, most studies have categorized cells into nine distinct types. These include immune cells (T cells, B cells, macrophages, plasma cells, neutrophils, and mast cells) and nonimmune cells (fibroblasts, endothelial cells, and epithelial cells). Among these, T cells, macrophages, and cancer-associated fibroblasts (CAFs) stand out as the most diverse and plastic cell populations within the TME. They play pivotal roles in angiogenesis, tumor cell migration, and antitumor immunity. These include immune cells (T cells, B cells, macrophages, plasma cells, neutrophils, and mast cells) and nonimmune cells (fibroblasts, endothelial cells, and epithelial cells). Among these, T cells, macrophages, and CAFs stand out as the most diverse and plastic cell populations within the TME. They play pivotal roles in angiogenesis, tumor cell migration, and antitumor immunity.

#### 4.2.1. Functional Dynamics of CD4^+^ and CD8^+^ T Lymphocytes in Antitumor Immunity

scRNA-seq has been used to delineate multiple functional subpopulations of classical T cells through gene expression analysis. To date, the majority of relevant studies have primarily focused on CD4^+^ and CD8^+^ T cells.

CD4^+^ T cells play complex and multifaceted roles in tumors, primarily by promoting tumor control through cytokine release and related helper cell functions [42]. Using scRNA-seq and high expression of relevant genes, Li et al. identified three subgroups of CD4^+^ T cells in samples from different stages of CC development and progression: naïve CD4^+^ T cells, regulatory T cells (Tregs), and central memory T cells. Among these, the Tregs in the SCC group showed high immunosuppressive activity and were associated with shorter survival. CD4^+^ T cells are one of the main components involved in the early immune response in metastatic lymph nodes (LNs) [28]. Additionally, Qiu et al. reported that Type 1 helper T cells (Th1-like CD4^+^ T cells) expressing *CCL4*, *GZMA*, and *GZMB* are involved in viral protein interactions, natural killer cell–mediated cytotoxicity, and T cell receptor signaling pathways, revealing their positive role in tumor immune defense [22].

CD8^+^ T cells are a key component of the TME and a fundamental element in cancer immunotherapy. Li et al. primarily analyzed the expression of relevant genes in CD8^+^ T cells, identifying three subgroups: naïve CD8^+^ T cells, cytotoxic CD8^+^ T cells, and exhausted CD8^+^ T cells. Naïve CD8^+^ T cells express markers such as *CCR7*, *KLF2*, *SELL*, and *LEF1*. Cytotoxic CD8^+^ T cells upregulate the expression of cytotoxic genes, such as *PRF1*, *GNLY*, *GZMA*, *GZMB*, and *GZMK.* In contrast, exhausted CD8^+^ T cells display increased expression levels of exhaustion-related molecules, such as *PDCD1 (PD-1)*, *CTLA4*, *LAG3*, and *HAVCR2 (TIM-3)*. Additionally, the authors used Monocle2 for pseudotime trajectory analysis, which indicated that CD8^+^ T cells in tumors undergo a process of exhaustion. This finding suggests that tumors exhibit an immunosuppressive state characterized by the infiltration of exhausted CD8^+^ T cells [28].

#### 4.2.2. Regulatory Roles of B Cells in Immune Modulation

Compared to T cells, B cells have received relatively less research attention. However, B cell signatures are often associated with favorable clinical outcomes in patients with CC [43]. Recently, there has been increasing recognition of the antitumor characteristics of B cells [44]. To investigate the role of B cells in CC, Cao et al. classified B cells into six subpopulations: activated B cells (marked by *TNFRSF13B* and *CD83*), memory B cells (marked by *BACH2* and *KLF4*), germinal center B cells (marked by *MME*, *AICDA*, and *BCL6*), plasma cells (further divided into PC_IGHA1 and PC_IGHG4 and marked by *MZB1* and *XBP1)*, and transitional B cells (also marked by *MZB1* and *XBP1*). Functional analysis revealed that genes specific to activated B cells were enriched in antigen processing and presentation pathways, while genes related to complement activation and immunoglobulin production pathways were enriched in plasma cells. This indicates distinct antitumor functions among different B cell subgroups [21]. Furthermore, B cells mainly originate from tumor samples rather than adjacent normal tissues, suggesting that a B cell response is elicited after stimulation within the TME [21].

#### 4.2.3. Polarization Dynamics of M1 and M2 Macrophages

In the TME, accumulating macrophages are referred to as tumor-associated macrophages (TAMs). Clinical data and extensive research suggest that TAMs generally exert procancer effects [45]. TAMs often exhibit different functional phenotypes, broadly categorized into M1 and M2 macrophages. M1 macrophages are typically associated with tumor-killing activities, primarily involved in antitumor responses and immune promotion. In contrast, M2 macrophages possess immunosuppressive properties and may facilitate tissue repair and tumor development [45–47]. TAMs exhibit functional plasticity, transitioning between proinflammatory M1 and immunosuppressive M2 phenotypes. Li et al. observed a shift from M1-dominant macrophages in precancerous lesions (high-grade squamous intraepithelial lesions, HSILs) to M2-dominant macrophages in invasive tumors. This shift correlates with immune evasion and disease progression. Paradoxically, macrophages associated with HSIL exhibited mixed pro- and anti-inflammatory signatures, reflecting early immune activation followed by suppression. Guo et al. further demonstrated that M2 polarization promotes malignant transformation, suggesting that targeting macrophage reprogramming (e.g., blocking M2-inducing cytokines like IL-10) could restore antitumor immunity [24]. These findings underscore the dual role of macrophages across different disease stages and their therapeutic relevance.

#### 4.2.4. CAFs in Stromal Remodeling

CAFs are key components of the tumor matrix and play a crucial role in facilitating interactions between cancer cells and the TME [48]. Numerous scRNA-seq studies have investigated the role of CAFs in tumor metastasis and their impact on prognosis, suggesting that CAFs may serve as potential therapeutic targets [12, 49].In their study of the TME in CC, Qiu et al. conducted a detailed investigation of CAFs, reclassifying them into two main types: inflammatory CAFs (iCAFs, characterized by high expression of *CKB*, *CFD*, and *DPT*) and myofibroblastic CAFs (myCAFs, characterized by high expression of *SULF1*, *INHBA*, and *COL8A1*). Further functional analysis revealed that iCAFs actively participate in the NFKB signaling pathway, complement and coagulation cascades, and cytokine–cytokine receptor interactions, highlighting their importance in promoting inflammation-mediated carcinogenesis in the development of CC [22]. Additionally, myCAFs not only play a crucial role in extracellular matrix remodeling but also significantly contribute to antigen processing and presentation [22]. In summary, both iCAFs and myCAFs promote CC progression through various pathways [22].

### 4.3. Mapping Evolutionary Trajectories of Cervical Carcinogenesis

Tumor occurrence and progression are multistage, complex, and dynamic evolutionary processes driven by multiple genetic alterations [50]. Understanding the evolution of the tumor ecosystem at each stage of cancer is crucial for early diagnosis, treatment, and risk assessment of malignant tumors [51]. scRNA-seq can identify genes or cell subpopulations that play a crucial role in tumor development and progression [52].

#### 4.3.1. Developmental Origins of Cervical SCC

To gain a deeper understanding of the occurrence and progression of CESC, Liu et al. employed scRNA-seq to analyze samples from 3 normal cervix (NC) tissue samples, 2 cervical intraepithelial neoplasia (CIN) samples, 3 early-stage CESC samples (International Federation of Gynecology and Obstetrics (FIGO) 2018 IB2 stage), and 5 locally advanced-stage CESC samples (FIGO2018 IIB to IIIC). As a proven marker highly expressed in normal cervical squamous cells, KRT14 showed high expression in NC samples but low expression in early and advanced CESC. The study revealed that its expression was high in NC samples but significantly reduced in both early- and locally advanced-stage CESC samples. In contrast, the expression of malignant squamous cell markers (*KRT17*) and typical proliferation markers (*CDKN2A*, *mki67*, and *TOP2A*) progressively increased from CIN to advanced-stage CESC, aligning with the tumorigenic process and progression of CESC [20]. In addition, the authors used Monocle analysis for trajectory inference and identified two distinct trajectories: one representing the tumorigenic trajectory of squamous cells and the other representing the developmental trajectory of nonmalignant squamous cells. The squamous cell subcluster Epi1, located at the beginning of the trajectory, was composed of *TP63KRT5++* basal stem cells. On the one hand, the Epi1 cluster may give rise to the malignant squamous cell subclusters Epi7 and Epi8, with increased expression of the malignant markers *MKI67* and *TOP2A*. On the other hand, Epi1 can also differentiate into the squamous cell subcluster Epi5, which shows more differentiated characteristics, including high expression of *SPRR3* and *SPRR2a*. Furthermore, cell cycle analysis of epithelial cells revealed that these epithelial cells were gradually activated along the tumorigenic trajectory, thereby further confirming the tumorigenesis of CESC [20].

#### 4.3.2. Molecular Transitions From Premalignant Lesions to Invasive Cancer

Guo et al. comprehensively mapped the transcriptional changes that occurred during the progression from normal epithelium to cancer induced by HPV. They discovered that three genes (*SPRR3*, *CEACAM7*, and *APOBEC3A*) were upregulated following HPV infection. These genes promoted viral persistence and genomic instability through their antiviral responses, effectively acting as “second hits” that accelerated the process of malignancy. In contrast, the downregulation of protective genes (*TCN1*, *TFF3*, and *BPIFB1*) in HSIL and cancer weakened the defenses against transformation. Additionally, six hub genes (*CALML5*, *CXCL10*, *KRT6C*, *KRTDAP*, *S100A7*, *SBSN*) were uniquely elevated during the transition from HSIL to cancer, serving as potential biomarkers for early detection and intervention [24]. This study provides a detailed molecular roadmap of cervical carcinogenesis and highlights actionable targets for intercepting disease progression.

**Figure 2:**
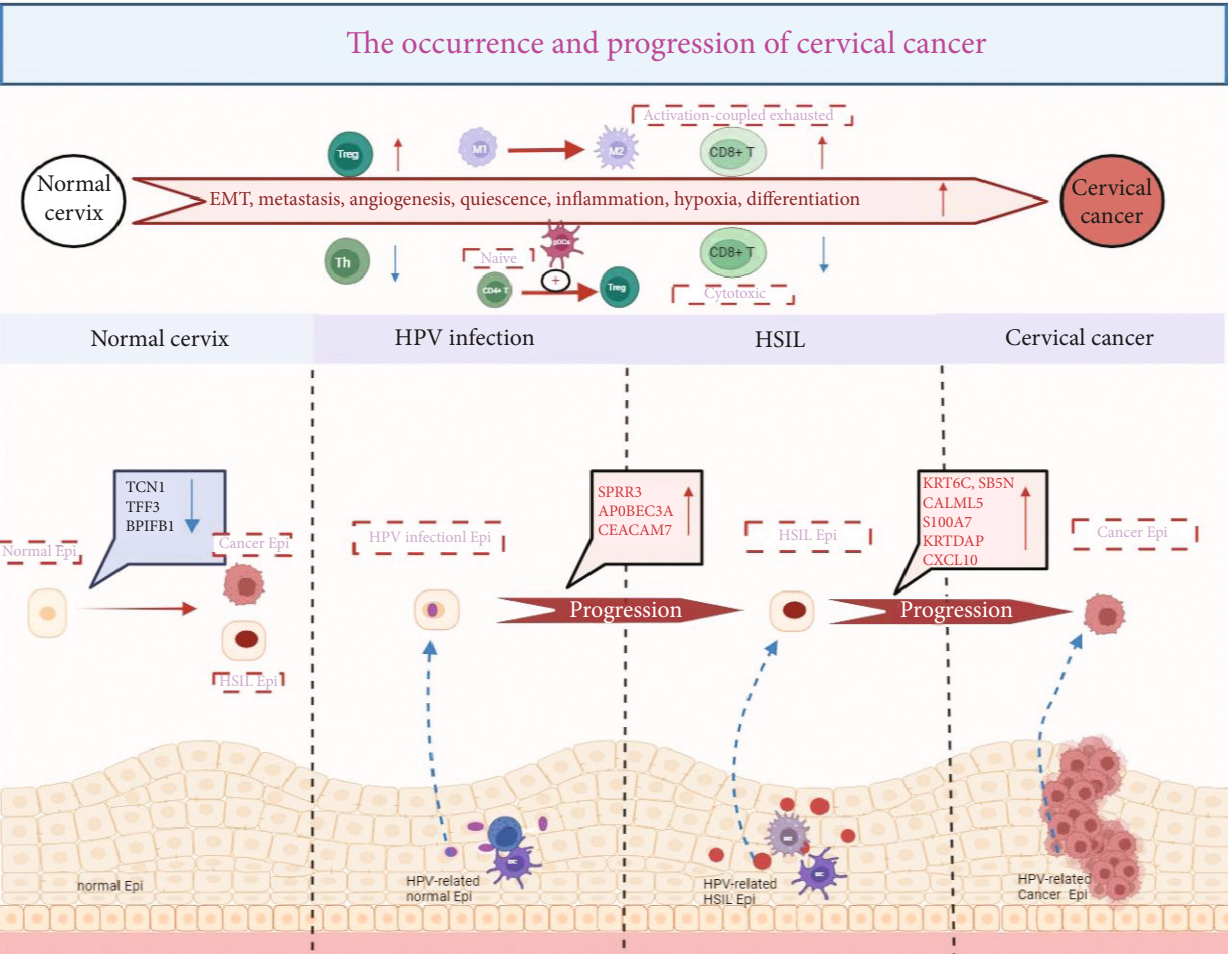
The occurrence and progression of cervical cancer. As the HPV-infected cervix progresses to precancerous lesions, three genes are upregulated (TCN1, TFF3, and BPIFB1), and three other genes are downregulated (SPRR3, CEACAM7, and APOBEC3A). Moreover, six genes were upregulated (CALML5, CXCL10, KRT6C, KRTDAP, S100A7, and SBSN) as precancerous lesions progressed to cancer. As the lesions progressed, macrophages polarized toward the M2 phenotype, and the activation of plasma cell-like DCs (pDCs) promoted the transformation of naïve CD4 T cells into Tregs. Furthermore, during the progression of normal cervix to cervical cancer, EMT, metastasis, angiogenesis, dormancy, inflammation, hypoxia, and differentiation gradually increase with lesion progression, with an increase in the abundance of Tregs and activation-coupled exhausted CD8^+^ T cells and a decrease in the abundance of Th and cytotoxic CD8^+^ T cells.

Unraveling the intricate processes of tumorigenesis and progression is crucial, as it not only helps identify potential therapeutic targets but also provides valuable biomarkers for diagnosis and prognosis. In our study, we meticulously mapped the mechanisms underlying the progression from a normal cervix to CC (Figure 2). Zhang et al. performed scRNA-seq on 122,400 cells derived from 20 cervical biopsy samples. These samples included 5 healthy controls, 4 patients with HSIL, 5 patients with microinvasive cervical cancer (MIC), and 6 patients with invasive cervical squamous cell carcinoma (ICC). By employing single-sample gene set enrichment analysis (ssGSEA) of tumor-associated features to compare the malignant cell clusters in HSIL, MIC, and ICC samples, they uncovered that key tumor-associated features, including EMT, metastasis, angiogenesis, dormancy, inflammation, hypoxia, and differentiation of malignant cells, progressively intensified with disease progression. Further investigation into the metabolic profiles of these cells revealed distinct metabolic signatures in the malignant cells of the HSIL, MIC, and ICC groups. Overall, these findings underscore the escalating heterogeneity and malignancy of tumor cells as the disease progresses [17]. Li et al. observed that HPV-infected epithelial cells no longer maintain the orderly arrangement typical of normal epithelial cells. This observation implies that the orderly cell arrangement is disrupted during the processes of cell differentiation, transformation, and cancer development. This study highlights the significant heterogeneity among cells in cancer tissues, which may exhibit unique genetic traits that distinguish them from normal epithelial cells [34]. Moreover, in an effort to investigate the heterogeneity of epithelial cells during the progression of CC, Liu et al. conducted scRNA-seq on 76,911 cells derived from 13 human cervical malignant tumor tissue samples at various stages. They identified eight distinct epithelial cell subclusters. Notably, Epi7 and Epi8—two epithelial cell subsets—showed specific enrichment in early- and late-stage CESCs and exhibited gene expression profiles associated with active tumor-related pathways, such as the cell cycle, DNA repair, and tumor invasion. Epi8, in particular, expressed genes related to potential immune suppression (indoleamine 2,3-dioxygenase 1, (*IDO1*)) and inflammation (*HLA-DRA*, *HLA-DPA1*, *IFIT*, and *CXCL9-11*), suggesting direct communication with T cells through ligand–receptor interactions [20].

#### 4.3.3. Metastatic Niche Adaptation and Immune Evasion Mechanisms

Tumor metastasis, as a crucial biological behavior, is one of the defining hallmarks of cancer [53]. Advanced CC is predominantly characterized by its invasive and metastatic capabilities, which are underpinned by alterations in a complex array of gene expression patterns. Unraveling the metastatic mechanism of CC is of paramount importance for gaining a comprehensive understanding of its development and for devising effective treatment strategies [54]. To investigate the cellular diversity and molecular characteristics underlying CC metastasis, Li et al. performed scRNA-seq analysis on a total of 5 samples from 4 CC patients, including 3 tumor tissues, 1 positive LN, and 1 negative LN. Their findings revealed distinct signaling pathway profiles between nonmetastatic and metastatic tumors. Specifically, nonmetastatic tumors exhibited significant enrichment in cell cycle signaling pathways, such as the G2M checkpoint, E2F target, MYC target V2, and mitotic signaling pathways. In contrast, metastatic tumors displayed upregulation of inflammation-related signaling pathways, including the IL6-JAK-STAT3 signaling pathway and pathways involved in coagulation and inflammatory responses. The authors found that, compared to nonmetastatic tumors, metastatic tumors exhibited a higher infiltration of immune cells, such as macrophages, neutrophils, and NK/T cells, while having fewer epithelial cells [29]. These findings suggest that once tumor metastasis occurs, tumor cells undergo a series of functional changes to adapt to the TME, facilitating immune escape. Moreover, tumor cells from primary tumors and metastatic LNs showed increased copy number variation (CNV) levels in different regions. Metastatic LNs exhibited an early activated TME, characterized by an increase in proliferative T cells, NK cells, and Tregs, as well as a decrease in cytotoxic CD8^+^ T cells and naïve T cells. The authors found that MRC1 expression in metastatic LNs was higher than that in metastatic tumors, leading them to conclude that a high percentage of C1QA + MRC1 macrophages might be associated with tumor metastasis. Additionally, they demonstrated that CAFs in metastatic LNs played prominent roles in antigen presentation, cell adhesion, and immune regulation. These results provide valuable insights into the mechanisms of CC metastasis and pave the way for new therapeutic approaches for CC [29].

### 4.4. Dissecting Mechanisms of Therapeutic Resistance in CC

Emerging evidence indicates that the development of drug resistance is a complex process, underpinned by a diverse array of cellular and molecular mechanisms. These mechanisms encompass genetic and epigenetic changes, cellular detoxification, and aberrant drug efflux and accumulation [32, 55, 56].

#### 4.4.1. Molecular Basis of Chemotherapy Resistance

Gu et al. conducted a detailed analysis of T cells, B cells, and myeloid cells using scRNA-seq and found that several signaling pathways closely associated with chemotherapy resistance, such as the PI3K/AKT and mitogen-activated protein kinase (MAPK) signaling pathways, were enriched across all three cell subpopulations. The PI3K/AKT signaling pathway plays a pivotal role in tumorigenesis, tumor progression, cell survival, and apoptosis. Meanwhile, the MAPK signal pathway, which regulates cell migration, survival, proliferation, and progression, was found to be significantly enriched and upregulated in patients resistant to chemotherapy. In addition, integrins and soluble factors secreted within the TME activate survival pathways involving PI3K/AKT and MAPK, resulting in the elevated expression of antiapoptotic proteins, thereby enhancing cell viability and contributing to drug resistance. The enrichment of the PI3K/AKT pathway in most differentially expressed genes (DEGs) suggests that the activation status of this pathway, which is commonly involved in tumor development, progression, and apoptosis, may be a key driver of chemoresistance in CC patients. Targeting specific components of the PI3K/AKT and MAPK pathways not only has the potential to enhance the efficacy of chemotherapy in CC but may also help overcome drug resistance [32].

#### 4.4.2. Cellular Adaptations Underlying Radiation Resistance

Radiotherapy is the primary radical treatment modality for patients with Stage IB3 to IVA CC and also represents an optional radical therapy for those with Stage IB1 to IB2 CC [3]. However, a significant challenge associated with radiotherapy is the potential development of radioresistance in cancer cells, rendering them less susceptible to ionizing radiation, which ultimately leads to treatment failure in cancer management [57].

Bi et al. used scRNA-seq to analyze three radioresistant CC samples. They found that radiation-resistant conditional reprogrammed (CR) cells, which exhibit radioresistance, predominantly clustered in the G1 phase of the cell cycle. In contrast, most radiation-sensitive cells, which are more susceptible to radiation, were found in the G2 phase. The study revealed that endoplasmic reticulum protein 29 (ERp29) plays a role in promoting endoplasmic reticulum stress–mediated radioresistance. Additionally, oxidative stress induces the monoubiquitination and transcriptional activity of FoxO4 in radioresistant cells, while simultaneously reducing the expression of the deubiquitinase *USP7*, thereby causing G0/S arrest and preventing radiation-induced cell death. Notably, the level of USP7 was decreased in radioresistant cells, whereas there was no significant difference in the expression of ERp29 [18].

### 4.5. Uncovering Potential Targets for CC

**Figure 3:**
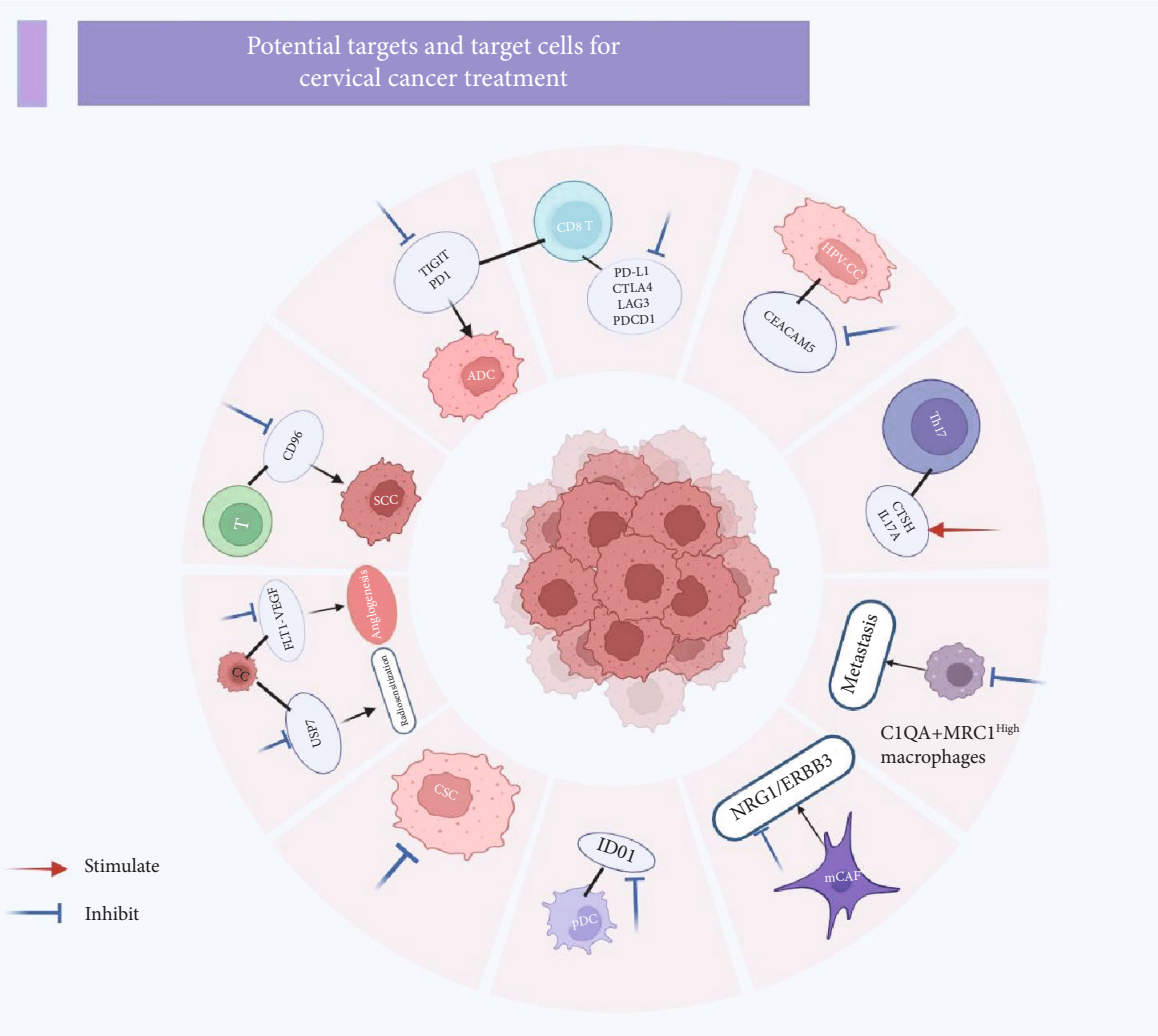
Potential target and target cells for cervical cancer treatment. Cervical cancer treatment involves a multifaceted approach targeting various cells and pathways. Targeted therapy focuses on blocking CEACAM5 on HPV-related cancer cells and inhibiting immune checkpoints (PD-L1, CTLA4, LAG3, and PDCD1) on CD8^+^ T cells. The NRG1/ERBB3 pathway and cancer stem cells (CSCs) are also targeted. Immunotherapy strategies include blocking TIGIT and PD-1 on CD8^+^ T cells for ADC and inhibiting CD96 on T cells for SCC. Radioresistance is addressed by targeting USP7 on cervical cancer cells. Antiangiogenesis is achieved by inhibiting FLT-1 and VEGF on cancer cells. Metastasis is prevented by targeting C1QA + MRC1^High^ macrophages. Immune modulation involves stimulating CTSH and IL-17A on TH17 cells to enhance antitumor immunity. (Red arrows represent stimulation, blue arrows represent inhibition, and black arrows indicate direction or movement.)

scRNA-seq technology offers a powerful approach to elucidate the characteristics and functions of distinct cell subsets by analyzing the gene expression profiles of individual cells. This detailed cellular and molecular information can significantly aid in the identification of potential therapeutic targets. The precision provided by single-cell sequencing not only accelerates the discovery of novel drug targets but also provides valuable insights into candidate drugs that can modulate the regulation of these targets, thereby potentially reducing drug development attrition [58].  Figure 3 summarizes the potential targets and target cells currently under investigation in the literature related to single-cell studies in CC.

## 5. ST in CC

Traditional bulk sequencing and histopathology often fail to capture intratumoral heterogeneity or spatial dynamics. For instance, while histopathology classifies CC into SCC and ADC subtypes, scRNA-seq revealed intermediate *KRT17*+ hybrid clusters with stem-like properties. ST further localized these cells to transition zones between glandular and squamous epithelia, suggesting a novel origin for mixed histology tumors [20, 27]. Similarly, ST resolved conflicting findings from bulk RNA-seq by showing that *PD-1*+ T cells in CC are not randomly distributed but are concentrated near *PD-L1*+ macrophage-rich regions. This spatial distribution explains why the efficacy of PD-1 blockade therapy varies across different areas of the tumor [21]. These insights, unattainable with conventional methodologies, directly inform therapeutic targeting strategies.

### 5.1. Revealing the Spatial Immune Microenvironment of CC

To investigate the spatial structure features of different cell subpopulations during the development trajectory of HPV-infected normal cervical epithelial cells to cancer cells, Guo et al. employed ST to analyze cervical tissue samples with varying HPV statuses and epithelial cell hyperplastic states. Their study encompassed a diverse set of samples: two HPV-negative normal samples, two HPV-positive normal samples, two HPV-associated HSIL samples, and three HPV-associated cancerous samples. They identified eight distinct regions within the cervical tissue: glands, normal squamous epithelium, connective tissue, HPV-infected epithelium, metaplastic squamous epithelium, HSIL lesions, and CC. The investigators mainly focused on immune cells, including myeloid cells and T cells, which typically surround epithelial cells, particularly in the context of CC. This approach allowed them to spatially map the tumor immune microenvironment. After establishing the spatial location of HPV-associated epithelial cells, they examined the spatial distribution of myeloid and T lymphocytes. Regarding CD8^+^ T cells, the study revealed that CD8^+^ exhausted T (Tex) cells infiltrated CC tissues more significantly. In addition, the spatial distributions of CD8^+^ Tex cells and HPV-associated cancer epithelial clusters in CC tissues were found to be consistent, suggesting that the interaction between these two cell subsets may mediate CD8^+^ T cell dysfunction and facilitate tumor immune escape. CD8^+^ mucosa-associated invariant T (MAIT) cells were highly abundant in HSIL tissues, including glands, connective tissues, and HSIL foci, where they played a crucial role in immune surveillance. Additionally, the researchers found that the abundance of CD4^+^ Tregs in CC tissues was greater than that in normal cervix and HSIL tissues. This finding underscores the immunosuppressive characteristics of the CC microenvironment [24].

### 5.2. New Targets for CC

ST is a cutting-edge technology that combines spatial information and transcriptomics, enabling the study of gene expression distribution and interactions within cells and tissues. This powerful tool facilitates the discovery of novel therapeutic targets. Guo et al. utilized ST to specifically analyze the spatial characteristics of gene expression in epithelial clusters and HPV-associated normal cervical tissue samples. They observed that the “HPV-related normal epithelial cluster” was highly abundant in HPV-positive normal cervix samples, with significant expression of *SLC5A8* and *DERL3*, mainly localized in the HPV-infected and metaplastic squamous epithelium. This finding highlights the spatial initiation of the HPV-induced malignant transformation. Furthermore, in HPV-associated HSIL tissue samples, “HPV-associated HSIL epithelial clusters” were prominently localized within HSIL lesions, characterized by high expression of *VSIG1* and *CASC9*. In addition, “HPV-associated cancer clusters” exhibited high expression of *CASP14* and *CALML5*. The specific high expression of these unique “HPV-associated epithelial cluster” genes provides a promising strategy for targeted therapy [24]. During the early stages of HPV infection, *CSF3* (HPV-associated normal epithelial cluster) and CSF1R/CSF3R (myeloid cells) are coexpressed within the HPV-infected squamous epithelium and metaplastic squamous epithelium, thereby triggering immune responses. In HSIL foci, spatial coexpression of *TNFSF10* (HPV-associated HSIL epithelial cluster) and *TNFRSF10B* (myeloid cells) was observed, mediating aberrant apoptosis and revealing immunosurveillance and clearance of precancerous aberrant epithelial cells. In CC tissues, several ligand–receptor pairs were spatially coexpressed, representing various aspects of cancer biology. For instance, *WNT7B* (CASP14, CALML5+ HPV-associated CA cluster) and *FZD1* (conventional dendritic cells (cDCs)) were coexpressed, indicating the aggressiveness of the cancer; *CEACAM5* (CASP14, CALML5+ HPV-associated CA cluster) and *CD1D* (cDC) were coexpressed, demonstrating tumor-associated antigen presentation; Furthermore, the coexpression of *TNFSF10* (CASP14, CALML5+ HPV-associated CA cluster) and *TNFRSF11B* (macrophage) revealed tumor escape through antiapoptosis, fully characterizing the malignant nature of the cancer. Collectively, these results provide a comprehensive spatial mapping of cellular interactions at different stages, from HPV infection to precancerous lesions and CC. They offer unprecedented insights into potential therapeutic targets at various disease stages [24].

## 6. Summary and Prospects

In conclusion, scRNA-seq and ST have revolutionized our understanding of CC by dissecting tumor heterogeneity at molecular, genetic, and epigenetic resolutions. For example, scRNA-seq has uncovered HPV-associated epigenetic silencing of tumor suppressors and activation of oncogenic pathways (e.g., Wnt/*β*-catenin), while ST has mapped the colocalization of genetically distinct clones with immune-suppressive niches. Future studies integrating multiomics data (e.g., single-cell ATAC-seq for epigenetics and whole-exome sequencing for mutations) will further elucidate the interplay between genetic drivers, epigenetic reprogramming, and TME dynamics, ultimately guiding precision therapies [6, 15, 24].

The integration of scRNA-seq and ST technologies has provided unprecedented insights into the complex landscape of CC. scRNA-seq has enabled the identification of distinct cell subpopulations and their functional states within the TME, revealing the intricate heterogeneity that underlies tumor progression, metastasis, and treatment resistance. Meanwhile, ST has complemented these findings by offering spatial context, demonstrating how different cell types and genetic clones are organized within the tumor ecosystem. This spatial information is critical for understanding how tumors establish immune-evasive niches and how therapeutic interventions might be optimized based on the tumor's spatial architecture.

Looking forward, the synergy between scRNA-seq and ST holds great promise for advancing CC research and treatment. The ability to simultaneously analyze genetic, transcriptional, and spatial features of tumors will facilitate the discovery of novel therapeutic targets and biomarkers. For instance, the identification of HPV-related genes and their spatial distribution could lead to targeted therapies that disrupt specific tumor–immune interactions. Additionally, the detailed characterization of immune cell subsets and their states may inform immunotherapeutic strategies, such as checkpoint inhibition or adoptive cell transfer, by highlighting which immune components are present and how they are regulated within the tumor.

Moreover, the combination of these technologies with other omics approaches, including proteomics and metabolomics, will provide a more comprehensive view of tumor biology. This multidimensional data integration will help address current limitations, such as the inability of scRNA-seq alone to fully capture the proteomic and epigenomic landscape of cancers. By incorporating spatial multiomics, researchers can more accurately differentiate tumor-enriched areas, nontumor regions, and tumor-infiltrated zones, thereby improving diagnostic accuracy and treatment planning.

Despite the high sample quality requirements, technical demands, limited throughput, and high costs associated with ST, rapid advancements in bioinformatics and sequencing technologies are poised to overcome these barriers. The era of single-cell multiomics, integrating genomics, transcriptomics, epigenomics, and proteomics, is imminent. This convergence will provide a holistic understanding of DNA, RNA, protein, and spatial heterogeneity, revolutionizing early detection, personalized therapy, and prognostic stratification.

In summary, the synergy of scRNA-seq and ST has redefined precision oncology in CC. By illuminating the molecular–spatial continuum of tumor ecosystems, these technologies empower researchers and clinicians to overcome the bottlenecks of conventional therapies, paving the way for innovative strategies to eradicate this global health burden.

## Figures and Tables

**Table 1:** Single-cell sequencing for the characterization of cervical cancer.

**Year**	**Author**	**Sample**		**Analysis target**	**Key conclusions**	**Potential targets or target cells**
		**Size**	**Type**			
2023	Zhang et al. [17]	20	5 N, 4 HSIL, 5 microinvasive carcinoma of the cervix, 6 invasive cervical squamous carcinomas	CSC, TME	Cervical CSCs originate from nonmalignant cervical stem cells in the normal cervix	CSCs

2023	Xing et al. [18]	5	3 radioresistant CC, 2 radiosensitive CC	Radioresistant	USP7 can be the potential radiosensitization targets in the future	*USP7*

2023	Chen et al. [19]	8	4 N, 4 CC	CD4^+^ T cells, M0 macrophages	Blocking the interaction between M0 macrophages and naïve CD4^+^ T cells may become a novel therapeutic strategy for cervical cancer immunotherapy	—

2023	Liu et al. [20]	13	3 N, 2 CIN, 3 early CESC, 5 advanced CESC	Cellular and molecular feature	This study contributes to an in-depth understanding of the initiation and progression of CESC, helping to refine the diagnosis of CESC and design optimal treatment strategies	mCAF-*NRG1/ERBB*3

2023	Cao et al. [21]	11	5 N, 6 CC	TIME	Large clonal cellular phenotypes and HPV antigen–specific T cell responses were revealed that might underlie the cornerstone of immunotherapy targeting T cells and/or HPV antigens	FLT1-VEGF

2023	Qiu et al. [22]	8	3 HPV SCC 3 HPV ADC 2 N-HPV ADC	TME	HPV16 stimulated much more clonal expansion than other high-risk HPV subtypes, such as HPV18 and HPV45, while the neoantigens in NHPVA ADC might also contribute to T cell proliferation and interaction; yet, there was an imbalanced immune response between CD8^+^ and CD4^+^ T cells	*TIGIT*, *PD1*

2023	Liu et al. [23]	13	5 1 week before RCT 5 3 weeks later RCT 3 N	*MHC-II*	Studying RCT-induced tumor ecosystems can help optimize and improve CC treatment	FCN1 M-MDSCs

2023	Guo et al. [24]	9 + 4	2 HPV-independent N, 2 HPV-associated N, 2 HPV-associated HSIL, and 3 HPV-associated CC Spatial transcriptome 4 cervical tissues from HPV-associated normal cervix, HPV-associated HSIL, HPV-associated SCC, and HPV-associated AC	TME, spatial immune microenvironment	Not only provide novel insights into HPV-related cervical carcinogenesis but also unprecedented possibilities for the accurate diagnosis, precise treatment and prognostic evaluation of CC	*CEACAM5*

2023	Fan et al. [25]	17 + 15	14 CC,3 N Spatial transcriptome 15 CC	TIME	Neoadjuvant chemotherapy induces a state transition to Epi-immune, which correlates with pathological complete remission following treatment with immune-checkpoint blockade	*FABP5*

2023	Ou et al. [26]	5 + 16	5 CC Spatial transcriptome 14 CC, 2 N	Cancer-associated fibroblasts, spatial transcriptomics, TME	The myofibroblasts may support the growth and metastasis of tumors by inhibiting lymphocyte infiltration and remodeling of the tumor extracellular matrix	Myofibroblasts

2023	Li et al. [27]	6	3 SCC, 3 ADC	Tumor heterogeneity, TME	Both SCC and ADC exhibit immunosuppressive TME	*IDO1*

2022	Li and Hua [28]	10	3 N, 2 HSIL, 4 CC, 1 metastatic lymph node	TME	HSIL exhibited a low, recently activated TME, tumor displayed immunosuppressive statue, and metastatic lymph node showed early activated phase of immune response	DCs *PD-1*, *PD-L1*, *CTLA4*

2022	Li et al. [29]	5	3 CC, 1 P-LN, 1 N-LN	The tumor ecosystems underlying cervical cancer metastasis	Provided a valuable resource for deciphering the comprehensive gene expression landscape of tumor and TME of primary and metastatic lesions in CC	C1QA + MRC1^high^ macrophages

2022	Li et al. [30]	2	2 HPV-related ADC	Cellular heterogeneity	Provide important insight into the cellular heterogeneity of HPV-related ADC and reveal key immune cell populations with tumor-suppressing functions	*FGFBP2*

2022	Li et al. [31]	6	3 CC, 3 NAT	Cellular heterogeneity	The hypoxia (S-H subtype) showed the worst prognosis, while CC patients of the immunoactive (S-I subtype) subtype had the longest overall survival time	*LAG3*, *TIM3*

2021	Gu et al. [32]	5	4 CC, 1 cervicitis	Chemotherapy resistance	Inhibition of certain components of the PI3K/AKT and MAPK pathways not only enhances chemotherapy efficacy in cervical cancer but also has the potential to overcome resistance	*PDCD1*, *LAG3*, *HAVCR2*

2021	Li et al. [33]	2	1 CC, 1 N	Intratumoral heterogeneity and transcriptional activities of ECs in CC	Tumor cells undergo a rapid transition from a naïve state to a stage of clonal expansion and activation	—

2023	Li et al. [34]	GSE168652		Cellular heterogeneity	Differences in HPV expression in different epithelial cell clusters suggest that HPV-infected epithelial cells exhibit dysregulation in cell differentiation and structural organization	*CKAP2L*

2023	Kang et al. [35]	GSE168652		Genes with AG features	Genes with AG features can effectively assess the prognosis of cervical cancer	*TXNDC12*

2022	Shen et al. [36]	GSE168652		EFNA1	EFNA1 may be a prognostic biomarker and potential therapeutic target in CC	*EFNA1*

Abbreviations: ADC, adenocarcinoma; AG, AGAMOUS; CC, cervical cancer; CESC, cervical squamous cell carcinoma; CIN, cervical intraepithelial neoplasia; CSC, cancer stem cell; HPV, human papillomavirus; N, normal cervix; NAT, normal adjacent tissue; NHPV, NO-HPV; RCT, radiotherapy; SCC, squamous cell carcinoma; TIME, tumor immune microenvironment; TME, tumor microenvironment.

**Table 2:** Cell clustering in cervical cancer.

**Year**	**Author**	**Cell clustering (markers)**	**Number of cells**
2023	Liu et al. [20]	T/NK (*CD2* and *CD3D*), B (*CD79A* and *MZB1*), Mye (*ITGAX* and *CSF1R*), Epi (*EPCAM* and *KRT5*), fibroblasts (*PDGFRA* and *RGS5*), ECs (*CDH5* and *KDR*)	76,911
2023	Cao et al. [21]	Epi (*EPCAM*), T (*CD3D*), NK (*KLRB1*), B (*CD79A*), PC (*CD38*), M (*FCN1*), DC (*CD14*), mast cells (*KIT*), ECs (*PECAM1*), fibroblasts (*COL1A1* and *COL12A1* for fibroblasts, *MCAM* and *ACTA2* for perivascular cells)	53,089
2023	Guo et al. [24]	Epi (*EPCAM*, *KLF5*), fibroblasts (*DCN*, *COL1A1*, *COL3A1*), ECs (*PECAM1*, *CDH5*, *VWF*) and smooth muscle cells (*ACTA2*, *RGS5*), Mye (*CD68*, *CSF1R*, *CD163*, *LYZ*), NK/T (*NKG7*, *CCL5*, *GZMA*, *CD3G*, *CD3E*, *CD3D*), NE (*NCF1*, *SORL1*), B (*CD19*, *BANK1*, *MS4A1*), mast cells (*TPSAB1*, *CPA3*), PC (*MZB1*, *IGHG1*, *IGKC*, *IGHG3*, *XBP1*, *JCHAIN*)	66,238
2023	Qiu et al. [22]	T (*CD2*, *CD3D*, and *CD3E*), B (*CD19*, *CD79A*, and *MS4A1*), PC (*IGHG1* and *TNFRSF17*), M (*CD14* and *C1QA*), NE/mast cells (*CSF3R/CPA3*), fibroblasts (*DCN* and *COL1A1*), smooth muscle cells (*TAGLN* and *ACTA2*), ECs (*PECAM1* and *VWF*), Epi (*EPCAM*, *KRT18*)	29,568
2023	Liu et al. [23]	Mye (*CD14* and *CD68*), T (*CD3D* and *CD3E*), PC (*SDC1* and *SLAMF7*), B (*MS4A1* and *CD79B*), DC (*CD1C*), ECs (*CDH5* and *VWF*), Epi (*EPCAM*), vascular fibroblasts (*RGS5* and *MYH11*), and matrix fibroblasts (*LUM* and *DCN*)	60,550
2023	Zhang et al. [17]	T, B, squamous Epi, columnar Epi, M, NE, mast cells, fibroblasts, ECs	122,400
2023	Wang et al. [41]	Malignant cells (*CDH1*, *EPCAM*, *CDKN2A*, *KRT14*, *KRT15*, and *KRT16)*, ECs (*PECAM1*, *EMCN*, and *CD34*), CD8^+^ T (*CD3D*, *CD3E*, *CD3G*, *CD8A*, and *NKG7*), M (*CD163*, *CD14*, and *MRC1*), cancer-associated fibroblast (*FAP* and *COL1A2*), smooth muscle cells (*ACTA2*, *CNN1*, *MYH11*, and *DCN*), vascular smooth muscle cells (*PDGFRB* and *MCAM*)	—
2023	Xing et al. [18]	T, B, squamous Epi, columnar Epi, M, NE, mast cells, fibroblasts, ECs	24,016
2023	Chen et al. [19]	T/NK, M, B cells, PC, mast cells, NE, ECs, smooth muscle cells, fibroblasts, stromal cells	87,248
2023	Li et al. [27]	Epi (*KRT8*, *CDKN2A*, *EPCAM*, *CDH1*), NK/T (*CD3D*, *NKG7*, *CD8A*), B (*CD19*, *MS4A1*, *CD79A*), NE (*CSF3R*, *FCGR3B*), Mye (*CD163*, *CD68*, *CD14*), mast cells (*MS4A2*, *KIT*), PC (*MZB1*, *IGHG4*), ECs (*PECAM1*, *EMCN*, *ENG*, *VWF*), fibroblasts (*COLIA1*, *DCN*, *ACTA2*, *TAGLN*)	61,723
2022	Kang et al. [35]	Epi, smooth muscle cells, M, fibroblasts, Lym, ECs	7303
2022	Li et al. [31]	T/NK cells, M, B cells, plasma cells, mast cells, NE, ECs, smooth muscle cells, fibroblasts	59,913
2022	Shen et al. [36]	Cancer cells (*CDH1*, *EPCAM*, *CDKN2A*), EC (*EGFL7*, *EMCN*, *PECAM1*), Lym (*CD28A*, *CD27*, *PRF1*), M (*CD163*, *FCGR2A*), fibroblasts (*COLIA2*, *APOD*), smooth muscle cells (*ACTG2*), endometrial stromal cells (*SUSD2*)	25,642
2022	Li et al. [29]	Epi (*CDKN2A*, *EPCAM*, *CD24*, and *CDH1*), ECs (*PECAM1*, *CDH5*, and *ENG*), fibroblasts (*COL1A2*, *DCN*, and *APOD*), smooth muscle cells (*ACTA2* and *ACTG2*), Lym (*CD3E*, *CD3D*, and *CD2*), M (*CD68*, *CD163*, *LYZ*), NE (*CSF3R*)	57,669
2021	Gu et al. [32]	T/NK cells, M, B cells, plasma cells, mast cells, NE, ECs, smooth muscle cells, fibroblasts	24,371

Abbreviations: DCs, dendritic cells; ECs, endothelial cells; M, macrophages; Mye, myeloid cells; NE, neutrophils; PCs, plasma cells.

## Data Availability

Data are available upon reasonable request from the corresponding author.

## Nomenclature

ADC: adenocarcinoma
AG: AGAMOUS
CAFs: cancer-associated fibroblasts
CC: cervical cancer
cDC: conventional dendritic cell
CESC: cervical squamous cell carcinoma
CIN: cervical intraepithelial neoplasia
CNV: copy number variation
CR: conditional reprogrammed
CSC: cancer stem cell
EMT: epithelial–mesenchymal transition
HSIL: high-grade squamous intraepithelial lesion
HPV: human papillomavirus
ICC: invasive cervical squamous cell carcinoma
IHC: immunohistochemistry
LN: lymph node
MAIT: mucosa-associated invariant T
MAPK: mitogen-activated protein kinase
MIC: microinvasive cervical cancer
NC: normal cervix
NAT: normal adjacent tissues
NHPV: NO-Human papillomavirus
pDC: plasmacytoid dendritic cell
RCT: radiotherapy
SCC: squamous cell carcinoma
scRNA-seq: single-cell RNA sequencing
ssGSEA: single-sample gene set enrichment analysis
ST: spatial transcriptomics
TAM: tumor-associated macrophages
TCR: T cell receptor
Th1: Type 1 helper T
TIME: tumor immune microenvironment
TME: tumor microenvironment
Tregs: regulatory T cells
